# British Medicinal Plants

**Published:** 1891-01

**Authors:** Alfred Heath


### BRITISH MEDICINAL PLANTS.

By Alfred Heath, M. D., F. L. S.

### Order 4.—Papaveraceæ (Continued).

*Chelidonium majus* (Celandine, Greater Celandine, Tetter-wort).—Found abundantly in many parts of this country, especially near villages or houses. Also in most parts of Europe as well as the United States. I have seen it in abundance growing around Philadelphia. This valuable drug was formerly used as an aperient and diuretic, and was recommended as a remedy in jaundice when not accompanied with inflammatory symptoms, but if not administered with caution it caused irritation of stomach and bowels. When collecting this plant I have been asked by country people if it is not a good thing for the liver. It was also used in dropsy and in cutaneous complaints. The fresh juice is used to destroy warts and diluted with milk to remove films in the eyes.

Jahr gives a proving of this drug, which, among other things, produces great costiveness, followed by nightly mucous diarrhœa, pressure on the bladder in the day-time with little discharge of water; also copious discharge day and night; pains and stiffness in right side of neck; cramp-like pain in right shoulder, hindering the motion of the arm; weariness and lassitude of the limbs; difficulty in moving the limbs quickly; dread of motion; yawning; feeling of drowsiness; great laziness after a meal, with drowsiness and indisposition to work; in the morning felt so weary could not get up.

### Order 5.—Fumariaceæ.

*Corydalis lutea* (Yellow Fumitory) on old walls.—There is no proving of this pretty little plant; it is probably similar in its action to the American plant, *Corydalis formosa*, of which there is a proving in Hale's *Materia Medica*.

*Fumaria officinalis* (Common Fumitory).—From *fumus*, smoke, because the juice, when dropped into the eye, produces the same sensation as smoke. It was formerly in esteem in many disorders of the skin of the leprous kind.

### Order 6.—Cruciferæ.

*Nasturtium officinalis* (*Sisymbrium nasturtium*) (Water-Cress, named nasturtium because the seeds when bruised irritate the nose).—This plant is known to every one, and is found in all parts of this country in water-courses, and is also extensively cultivated as a salad. Its virtues are many. It is officinal in the French pharmacopœia, and is used in various affections of the skin; it is given as a remedy in certain forms of cancer; it is an ingredient in nostrums for the cure of cancer. There is no "proving" of this drug; the tincture made from the plant when in flower and seed may be had of any homœopathic chemist.

*Sisymbrium officinale* (*Erysimum officinale*) (common name, Hedge Mustard).—Found on every roadside during the summer. There is no proving, but the plant has been recommended as a remedy in fever, and is sometimes an ingredient in nostrums for the cure of fevers. It was formerly used as an expectorant, and also on account of its diuretic properties.

*Brassica oleracea* (The Wild Cabbage, Sea Cabbage).—Found on sea-cliffs in the south and west of England. This plant is said to be the origin of all our garden cabbages, however different their appearance. In its cultivated form cabbage is considered a very wholesome article of diet, but it is apt to produce flatulency, especially in persons of weak digestion. I have known cases in which it invariably produced *ascarides*, which were apparently not present before. In its mild form, when prepared as a drug, it has been recommended in scrofulous diseases.

*Sinapis nigra* (Black Mustard).—Found on river banks and banks of ditches. The plant is cultivated largely in Essex and from its seed mustard is made. It is stated that in the Island of Ely wherever new ditches are thrown out, or the earth dug to any unusual depth, a crop of black mustard immediately appears, the seeds having remained under ground probably for ages. Its acrimony is due to an essential oil. *Moult arde*, in old French (it burns much) might have been imagined the real meaning of the word mustard had not a whimsical history attached to its etymology. In 1382, Philip the Bold, Duke of Burgundy, granted to the town of Dijon armorial ensigns with the motto, "Moult me tarde" (I long or wish ardently), which, being sculptured over the principal gate, by some accident the middle word became effaced. The merchant dealers in Sinapis intending to ensign their pots with labels bearing the city arms, copied the imperfect motto as it then remained, "*Moult-tarde*," and hence the name mustard (*Cyclopædia of Botany*). *Sinapis nigra* has been used empirically in disorders of the respiratory organs and kidneys, in dropsy, and in rheumatism. Mustard water is commonly used as an emetic. There is a proving of this drug in Dr. T. F. Allen's *Hand-book of Materia Medica*. It produces among other things hoarseness in the evening, with constant attempts to clear the throat; hacking cough with expectoration of lumps of mucus, the cough generally beginning about seven or eight P. M. Pain in the bladder, frequent, *copious* urination, day and night; rheumatic pains in intercostal and lumbar muscles, worse toward night; sleeplessness from pain in back and hips; chilliness, fever, heat down the spine, as from hot water, also with sweat on the forehead and upper lip.

*Sinapis alba* (White Mustard).—Found on cultivated and waste calcareous lands. This plant has also been mentioned from time to time. Its action is probably similar to the *S. nigra*. There is no proving.

